# Rationally Designed Anti-CRISPR Nucleic Acid Inhibitors of CRISPR-Cas9

**DOI:** 10.1089/nat.2018.0758

**Published:** 2019-05-30

**Authors:** Christopher L. Barkau, Daniel O'Reilly, Kushal J. Rohilla, Masad J. Damha, Keith T. Gagnon

**Affiliations:** ^1^ Department of Biochemistry and Molecular Biology, School of Medicine, Southern Illinois University, Carbondale, Illinois.; ^2^ Department of Chemistry, McGill University, Montreal, Canada.; ^3^ Department of Chemistry and Biochemistry, Southern Illinois University, Carbondale, Illinois.

**Keywords:** anti-CRISPR, RNA, CRISPR-Cas9, inhibition, nucleic acid, gene editing

## Abstract

Clustered regularly interspaced short palindromic repeat (CRISPR) RNAs and their associated effector (Cas) enzymes are being developed into promising therapeutics to treat disease. However, CRISPR-Cas enzymes might produce unwanted gene editing or dangerous side effects. Drug-like molecules that can inactivate CRISPR-Cas enzymes could help facilitate safer therapeutic development. Based on the requirement of guide RNA and target DNA interaction by Cas enzymes, we rationally designed small nucleic acid-based inhibitors (SNuBs) of *Streptococcus pyogenes* (*Sp*) Cas9. Inhibitors were initially designed as 2′-*O*-methyl-modified oligonucleotides that bound the CRISPR RNA guide sequence (anti-guide) or repeat sequence (anti-tracr), or DNA oligonucleotides that bound the protospacer adjacent motif (PAM)-interaction domain (anti-PAM) of *Sp*Cas9. Coupling anti-PAM and anti-tracr modules together was synergistic and resulted in high binding affinity and efficient inhibition of Cas9 DNA cleavage activity. Incorporating 2′F-RNA and locked nucleic acid nucleotides into the anti-tracr module resulted in greater inhibition as well as dose-dependent suppression of gene editing in human cells. CRISPR SNuBs provide a platform for rational design of CRISPR-Cas enzyme inhibitors that should translate to other CRISPR effector enzymes and enable better control over CRISPR-based applications.

## Introduction

The discovery of gene editing and programmable genomic control by clustered regularly interspaced short palindromic repeat (CRISPR) RNAs (crRNAs) and their CRISPR-associated (Cas) proteins [1–5] holds tremendous promise for future therapeutics and curing genetic diseases [6–11]. Despite their potential, CRISPR enzymes are not optimal for therapeutic applications [9,10,12–14]. For example, they invariably suffer from unwanted “off-target” editing that can be difficult to predict, detect, or prevent [12,15–19].

The safety of CRISPR in human patients will remain an important hurdle for practical drug development [7,19–22]. To fully implement CRISPR-based therapeutics, it may be necessary to develop kill-switch inhibitors that can halt activity on demand. Several therapeutic drugs have benefitted from development of antidotes to counter unexpected side effects and protect patients. For example, vitamin K and prothrombin complex concentrate are used as antidotes to reverse adverse reactions or overdoses from anticoagulants like warfarin [23], and protamine sulfate reverses anticoagulation by heparin [24].

Engineered Cas variants have been previously designed to respond to light or small molecules to control activity [25,26]. However, these systems are engineered for CRISPR-Cas9 activation rather than efficient and rapid inhibition. Turning off activity in these systems is dependent on relatively slow protein dissociation or protein turnover kinetics. Recently, natural anti-CRISPR proteins have been found in bacteriophage genomes that can shut down CRISPR systems by inhibiting DNA binding or blocking conformational states required for catalysis [27–30]. Recent characterization of natural anti-CRISPR proteins provides inspiration for design of biologic drugs that may mitigate potential problems with CRISPR-based therapeutics [31]. The reliance of Cas enzymes on RNA guides and the growing success of nucleic acid therapeutics suggest that nucleic acids may serve as a viable platform for designing inhibitors with drug-like properties.

Using existing structural data and the principles of nucleic acid pairing and protein interaction [29,32,33], we rationally designed nucleic acid-based inhibitors of *Streptococcus pyogenes* (*Sp*) Cas9. Our approach utilizes small, chemically modified anti-CRISPR nucleic acids comprising modules that interact with *Sp*Cas9 ribonucleoprotein (RNP) at the repeat sequence of the guide RNA and the protospacer adjacent motif (PAM)-interacting (PI) domain of *Sp*Cas9. This approach resulted in anti-CRISPR nucleic acids that efficiently bound *Sp*Cas9 RNPs with high affinity and inhibited catalytic activity *in vitro* and inside of cells. Anti-CRISPR nucleic acids represent a novel class of inhibitors capable of stopping CRISPR activity and facilitating safer implementation of CRISPR-based therapeutics.

## Materials and Methods

### RNA and inhibitor synthesis

**SCHEME 1.**
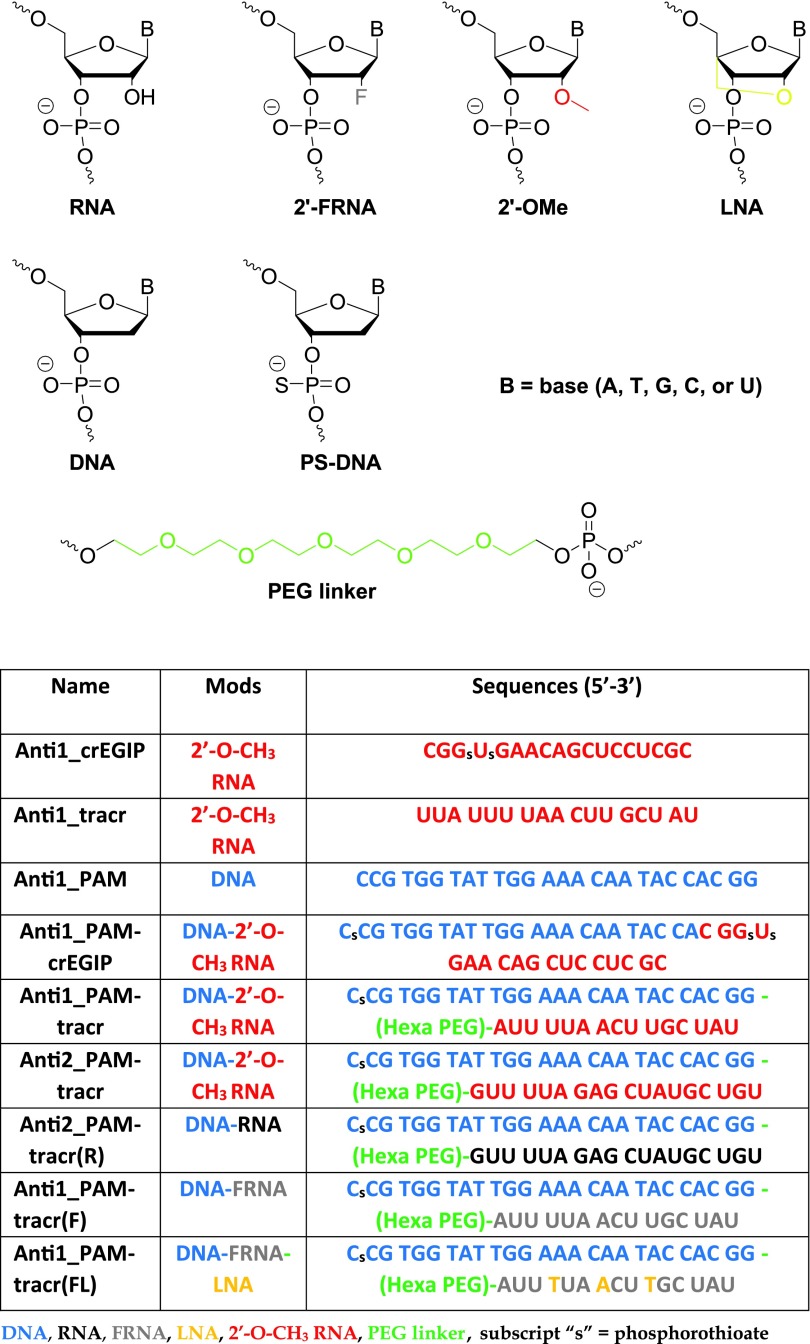
Chemical modifications and nucleic acid-based inhibitor modification schemes used in this study. LNA, locked nucleic acid; PAM, protospacer adjacent motif; PEG, polyethylene glycol; PS, phosphorothioate.

Inhibitor molecules and crRNAs were commercially synthesized by Integrated DNA Technologies (IDT) or custom synthesized (Scheme 1; Supplementary Table S1). Custom synthesis used standard phosphoramidite solid-phase conditions. Syntheses were performed on an Applied Biosystems 3400 or Expedite DNA Synthesizer at a 1 μM scale using Unylink CPG support (ChemGenes). All phosphoramidites were prepared as 0.13 M solutions in acetonitrile (ACN), except DNA, which was prepared as 0.1 M solutions. 5-Ethylthiotetrazole (0.25 M in ACN) was used to activate phosphoramidites for coupling. Detritylations were accomplished with 3% trichloroacetic acid in CH_2_Cl_2_ for 110 s. Capping of failure sequences was achieved with acetic anhydride in tetrahydrofuran (THF) and 16% *N*-methylimidazole in THF. Oxidation was done using 0.1 M I_2_ in 1:2:10 pyridine:water:THF. Coupling times were 30 min for 2′F-RNA, and 50 min for locked nucleic acid (LNA) phosphoramidites. Deprotection and cleavage from the solid support were accomplished with 3:1:0.2 NH_4_OH:EtOH:DMSO at 65°C for 16 h. Crude oligonucleotides were purified by anion-exchange high performance liquid chromatography (HPLC) on an Agilent 1200 Series Instrument using a Protein-Pak DEAE 5PW column (7.5 × 75 mm) at a flow rate of 1 mL/min. The gradient was 0%–24% solution 1 M LiClO_4_ over 30 min at 60°C. Samples were desalted on NAP-25 desalting columns according to manufacturer protocol. Modified crRNAs were prepared for RNP assembly by heating to 95°C and then placing on ice to prevent formation of stable secondary structures.

TracrRNA and single-guide RNA (sgRNA) were prepared by T7 *in vitro* transcription with DNA templates synthesized by IDT (Supplementary Table S1). Single-stranded DNA templates were annealed to T7 promoter oligo to generate double-stranded promoter regions, which support *in vitro* transcription by T7 RNA polymerase. Transcription reactions were performed by standard protocols for 2 h. Briefly, reactions contained purified T7 RNA polymerase, 30 mM Tris (at pH 7.9), 12.5 mM NaCl, 40 mM MgCl_2_, 2% polyethylene glycol (PEG) 8,000, 0.05% Triton X-100, 2 mM spermidine, and 2.5 μM T7-DNA template. Afterward, the DNA template was degraded by the addition of 1 U of DNase I for every 20 μL of reaction and incubated at 37°C for 15 min. Reactions were phenol-chloroform extracted and gel purified from denaturing polyacrylamide gels. Purified RNA was quantified by measuring absorbance at 260 nm and calculated extinction coefficients using nearest neighbor approximations and Beer's Law.

### Preparation of *Sp*Cas9 and “Dead” *Sp*Cas9 (dCas9)

Plasmid encoding an *Sp*Cas9 with a C-terminal fusion of a nuclear localization signal (NLS) and a 6x-Histidine tag (pET-Cas9-NLS-6xHis) was obtained from Addgene (62933). A dead Cas9 (dCas9) version was prepared by performing site-directed mutagenesis on this plasmid to generate H840A and D10A mutations (pET-dCas9-NLS-6xHis).

Protein expression was induced in Rosetta (DE3) cells with 0.4 mM IPTG at 18°C for 16 h. Cell pellets were resuspended in 6 mL of chilled binding buffer (20 mM Tris-HCl, pH 8.0, 250 mM NaCl, 1 mM PMSF, and 5 mM imidazole) per 0.5 L of culture. Resuspended cells were sonicated and clarified by centrifugation. His-Pur Cobalt-CMA resin (Thermo Scientific) was equilibrated with binding buffer and the supernatant added to the equilibrated resin and incubated at 4°C for 1 h. The supernatant was washed sequentially with increasing concentrations of NaCl in 50 mL volumes of wash buffer (Tris-HCl, pH 8, 0.25/0.5/0.75/1.0 M NaCl, and 10 mM imidazole). Protein was eluted with 15 mL elution buffer (Tris-HCl, pH 8, 250 mM NaCl, and 130 mM imidazole). Purified Cas9 was concentrated with Vivaspin 15 centrifugal concentrators (Sartorius, 30K molecular weight cut-off [MWCO]). Concentration was approximated by ultraviolet (UV) absorbance at 280 nm using a calculated extinction coefficient (120,450 M-1 cm-1) and Beer's law [34]. One volume of glycerol was added to a final of 50% and purified Cas9 stored as aliquots at −80°C.

### Radiolabeling of RNA, DNA, and nucleic acid-based inhibitors

The 5′ phosphate on T7-transcribed tracrRNA was removed using alkaline phosphatase following the manufacturer's recommended protocol. Synthetic duplex target DNA and crRNA lacks a 5′ phosphate and was directly labeled. A total of 100 pmols of tracrRNA, crRNA, or antisense DNA target strand was radiolabeled with [γ-32P]-ATP using T4 polynucleotide kinase following the manufacturer's recommended enzyme protocol. Reactions were phenol-chloroform extracted and radiolabeled RNA or DNA was gel purified on 15% denaturing polyacrylamide gels (1× Tris-borate ethylenediaminetetraacetic acid (EDTA) (TBE), 7 M urea) by the crush-and-soak method. Gel-purified radiolabeled RNA and DNA were quantified by scintillation counting.

### Determining the active concentration of Cas9 and dCas9

The active concentration of Cas9 and dCas9 proteins was determined by titration of increasing amounts of Cas9 or dCas9 with 0.5 μM crRNA:tracrRNA complex, where 500 cpms of radiolabeled crRNA was spiked into the reaction. Cas9 or dCas9 binding to crRNA:tracrRNA complex was determined by dot-blot filter binding assays. At concentrations above the *K*_d_ value, binding is proportional to the amount of protein added and results in a straight line when plotting radioactivity versus protein. Once Cas9 or dCas9 binding has saturated the crRNA:tracrRNA ligand, binding plateaus and is also a straight line. The value of *x* where the two lines intersect is equivalent to 0.5 μM of Cas9 or dCas9. To find this value, the two linear equations were set equal to one another and algebraically solved for *x*.

### Dot-blot filter binding assays

For inhibitor binding to Cas9 RNP complexes, radiolabeled inhibitor (500 cpms/reaction) was combined with increasing concentrations of a preassembled dCas9-tracrRNA complex, with or without crRNA bound, in a final reaction of 40 μL 1× cleavage buffer (20 mM Tris-HCl, pH 7.5, 100 mM KCl, 5% glycerol, 1 mM DTT, 0.5 mM EDTA, and 2 mM MgCl_2_) and 0.1 mg/mL of purified yeast transfer RNA (tRNA). After incubation at 37°C for 15 min, reactions were vacuum filtered over nitrocellulose membrane (Protran Premium NC, Amersham) using a 96-well dot-blot apparatus. Wells were washed twice with 200 μL of 1× cleavage buffer. Membrane was then removed and washed with 1× phosphate buffered saline (PBS) solution thrice for 15 min and air dried at room temperature (RT). Binding of radioactive crRNA was then visualized by phosphorimager on a Typhoon FLA 9500. Spots were quantified with ImageQuant software, plotted in Prism (GraphPad) and fit to a one-site binding hyperbola equation. Error bars for all quantified data represent experimental replicates, not technical replicates. Sample size was selected based on the expectation that two or more separate replicates will be representative of typical *in vitro* assay conditions.

### Gel-shift assays

dCas9-tracrRNA complexes were prepared at room temp in 1× Cas9 cleavage buffer supplemented with 5 μg bovine serum albumin (BSA), 5 μg yeast tRNA, and 10U SUPERase-in (ThermoFisher). Complex was titrated onto radiolabeled crRNA (5,000 cpm/reaction) in 1× Cas9 cleavage buffer in 20 μL reactions. Reactions were incubated at 37°C for 15 min. Resultant RNP complexes were then resolved on a TBE-buffered 6% native polyacrylamide gel at 4°C. For tracrRNA-crRNA gel shifts, dCas9 was omitted from the binding reactions. Gels were vacuum dried and exposed to phosphorimager screen and visualized with a Typhoon FLA 9500. Gel shifts were quantified using ImageJ software, plotted in Prism (GraphPad), and fit to a one-site binding hyperbola equation, and *K*_d_ values calculated by nonlinear regression.

### *In vitro* Cas9 cleavage activity assays

Linearized enhanced green fluorescent protein (EGFP)-encoding plasmid or PCR-amplified DNA (1 kB fragment, pEGIP primers in Supplementary Table S1) from an alternative EGFP-containing plasmid (pEGIP, Addgene No. 26777) was used as target DNA substrates and prepared as previously described [35,36]. The Cas9 pre-RNP complex was assembled (typical final concentrations: 0.75 μM Cas9 and 0.25 μM tracrRNA) in a 1× cleavage buffer supplemented with 0.1 mg/mL of purified yeast tRNA. The concentration of tracrRNA was purposely set as the limiting component of the RNP complex and used to predict final RNP concentration. Inhibitors at the indicated concentrations were then combined with Cas9-tracrRNA at 37°C for 15 min to assemble an inhibitory complex. The crRNA (typically 0.3 μM final) and target DNA (100 ng) spotted into tubes. The Cas9-tracrRNA-inhibitor complex was then added to these tubes to begin the reaction. A small molar excess of Cas9 and crRNA helps ensure complete assembly of tracrRNA into RNP complexes. Inhibitor molecules were added at the final concentrations indicated in each experiment. For time-course experiments, reactions were as described above, except 0.5 μM tracrRNA was used and crRNA and inhibitor concentrations were set at 1 μM to ensure that tracrRNA and Cas9 were fully assembled into complexes. Under these conditions, the crRNA must compete with the inhibitor for binding to Cas9-tracrRNA.

Standard reaction conditions were 37°C for 10 min or up to 1 h for time courses in a final reaction volume of 40 μL. The reaction was stopped by the addition of 10 volume of 2% LiClO_4_ in acetone and precipitated for >1 h at −20°C. Precipitated reactions were centrifuged and washed with acetone, air dried, and resuspended in 1× loading dye (10% glycerol, 1× TBE, orange G dye) containing 10 μg of Proteinase K. For time-course experiments, reactions were stopped at specified time points by the addition of 2% LiClO_4_ in acetone and placed on ice, and then worked up the same as other samples. After dissolving the pellet, the reactions were incubated at RT for 20 min, and then resolved on TBE-buffered 1% agarose gels. Gels were stained with ethidium bromide and visualized by UV imager.

The fraction of target cleaved was quantified using ImageJ software. The band intensity for the cleavage product band was divided by the combined intensity of cleavage product and uncut substrate bands and reported as fraction cleaved (ie, “cut”/”cut + uncut”). Time course cleavage assay results were plotted using Prism (GraphPad) software and fit to a one-site binding hyperbola by nonlinear regression. Error bars for all quantified data represent experimental replicates, not technical replicates. Sample size was selected based on the expectation that three or more replicates will be representative of typical *in vitro* assay conditions.

### Cell-based editing measured by flow cytometry

HEK 293T cells expressing EGFP alone or EGFP and *Sp*Cas9 were a kind gift from Wen Xue (UMass Medical Center) [37]. Cells were grown in Dulbecco's modified Eagle's medium with 1× non-essential amino acids, 5% Cosmic calf serum, 2.5% fetal bovine serum (FBS), and without antibiotics. Cas9-expressing cells were reverse transfected (40–50,000 cells) in six replicates in 96-well plates with 20 pmols of sgRNA or crRNA:tracrRNA complex in a final of 200 μL using RNAiMAX (Invitrogen) following the manufacturer's recommended protocol. Inhibitors were co-transfected with guide RNA (20 pmols) at a 0.5:1, 1:1, 2:1, or 4:1 molar ratio of inhibitor to guide RNA. After 12 h, 1 volume of media containing 15% FBS and 1× Penicillin-Streptomycin solution was added to the Opti-MEM and cells incubated for an additional 12 h. Media were then replaced with full media and cells grown for an additional 4 days with fresh medium change every 2 days.

In the case of cells expressing only EGFP, assembled Cas9 RNP and inhibitors were co-electroporated into cells using the 10 μL Neon Electroporation System (Invitrogen) in six replicates. This experiment (sufficient for 6.5 replicates) was prepared as follows: reactions containing 0.9 μL sgRNA (72.5 μM), 2.64 μL inhibitor (100 μM), and 0.85 μL *Sp*Cas9 (8 μM effective concentration) were preincubated for at least 5 min at room temperature while cells were prepared. When necessary, as for the no inhibitor control and intermediate concentrations, the inhibitor was diluted or replaced with an unrelated nontargeting RNA (100 μM). A total of 260,000 cells were aliquoted in a microcentrifuge tube for each condition before being centrifuged at 1000*g* for 1 min. The cells were then washed with 1× PBS and centrifuged again, and the PBS removed; 1.5 μL carrier DNA (100 μM) and 72.1 μL buffer R were added to the equilibrated Cas9 RNP and inhibitor mix and used to resuspend the cell pellet. Ten microliters of this was taken into an electroporation tip and electroporated with the following program: 1,150 V pulse, pulse width of 20 s, and 2 pulses. The electroporated cells were then added to a well in a 96-well dish with 190 μL of full media. This was repeated five times for a total of six replicates. Cells were cultured under normal conditions for 5 days before flow cytometry.

For flow cytometry, cells were washed with PBS, trypsinized, washed again, and then fixed with 1% paraformaldehyde in PBS for 8 min. Cells were washed again and counted in an Accuri C6 Flow Cytometer. EGFP was detected using the blue laser at excitation 488 nm; emission detection 530 ± 15 nm (FL1 channel). At least 15,000 events were collected and analyzed by Accuri CFlow Plus software. The cells were first gated based on forward and side scattering (FSC-A/SSC-A) to remove cell debris, and then gated to select single cells (FSC-H/FSC-A). Finally, cells were gated to select EGFP positive cells. The quadrant gate was based on the signal from non-EGFP expressing control cells. Untreated HEK 293T cells expressing EGFP and *Sp*Cas9 contained ∼6% nonfluorescent cells. The average from three replicates was used for background subtraction to determine the extent of cell-based editing after treatment.

## Results

**FIG. 1.**
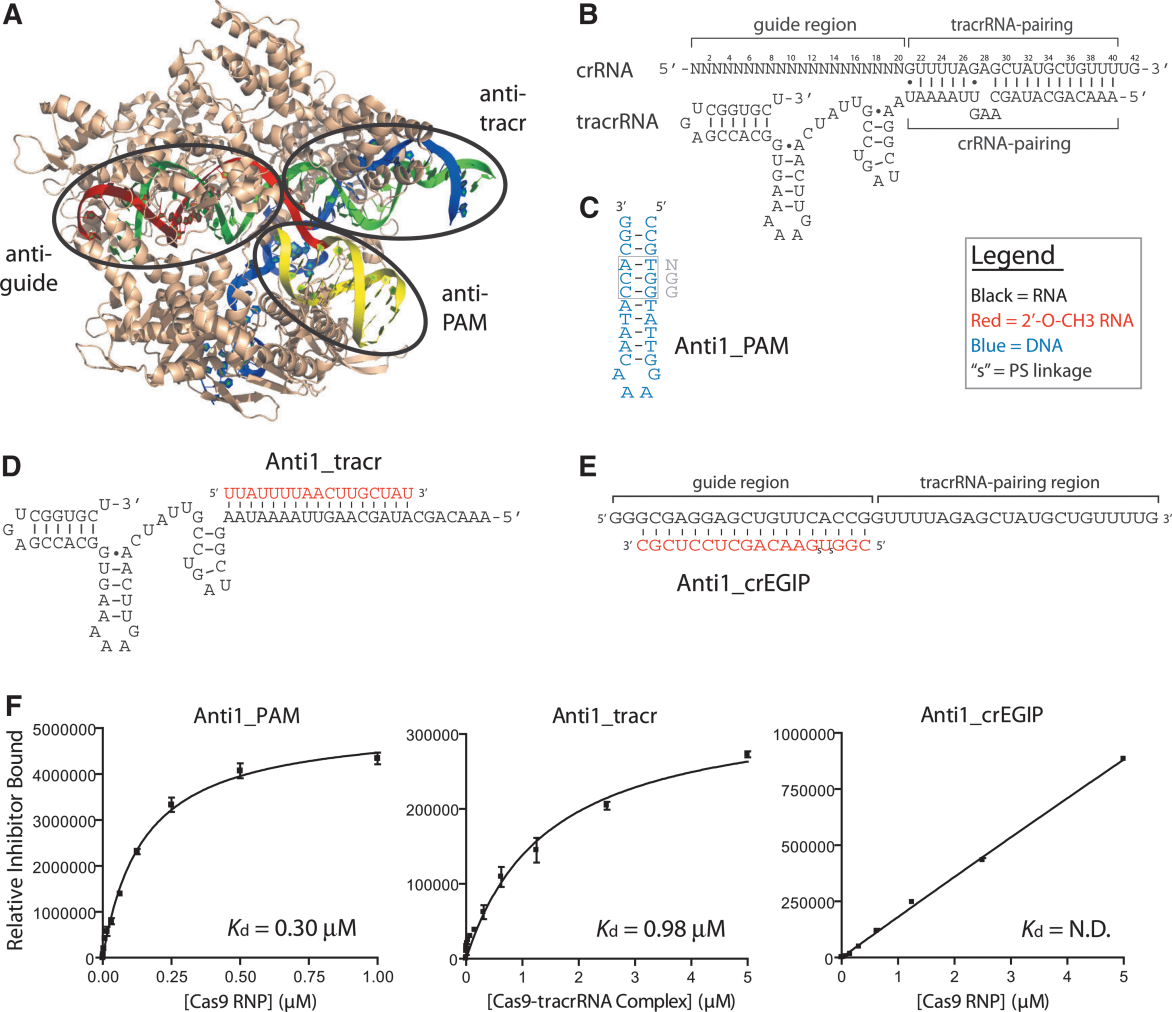
Nucleic acid-based inhibitor module design and activity. **(A)** Points of contact for nucleic acid-based inhibitors of CRISPR-*Sp*Cas9 [33]. *Sp*Cas9 protein is depicted in *brown*, tracrRNA in *blue*, crRNA in *green*, complementary DNA target in *red*, and PAM-containing DNA in *yellow*. **(B)** Illustration of a dgRNA for CRISPR-*Sp*Cas9. sgRNA (not shown) consists of a shorter crRNA-tracrRNA pairing stem that is closed by a GNRA tetraloop [2]. (**C**–**E**) Sequence, secondary structure, and pairing of nucleic acid-based inhibitor modules. **(F)** Binding curves and calculated affinity of inhibitor modules for the *Sp*Cas9-tracrRNA or *Sp*Cas9-tracrRNA-crRNA RNP complexes determined by *dot*-blot filter binding. Error bars are standard error of the mean (SEM). CRISPR, clustered regularly interspaced short palindromic repeat; dgRNA, dual-guide RNA; PAM, protospacer adjacent motif; PS, phosphorothioate; RNP, ribonucleoprotein; SEM, standard error of the mean; sgRNA, single-guide RNA.

To investigate the potential of nucleic acids as inhibitors for *Sp*Cas9, we designed and synthesized chemically modified candidate inhibitors (Scheme 1). Inhibitor modules were designed to compete with a target DNA sequence (anti-guide), the repeat sequence of the crRNA that pairs to *trans*-activating crRNA (tracrRNA) (anti-tracr), or the PAM motif and flanking duplex DNA of a target (anti-PAM) (Fig. 1A, B). We reasoned that inhibitors would likely need to bind at equal or greater affinity to *Sp*Cas9 than the crRNA, tracrRNA, or target DNA to provide reasonable competition. Thus, we first chose to investigate binding affinity of inhibitor candidates for *Sp*Cas9 RNP complexes. Before performing inhibitor binding studies, we also quantified the active concentration of *Sp*Cas9 by titrating its binding to a radiolabeled crRNA-tracrRNA complex by dot-blot analysis (Supplementary Fig. S1A). This method works well for RNA binding proteins where binding stoichiometry is known and binding affinities can be approximated. It avoids inconsistencies in standard protein quantification techniques and ensures accurate apparent binding affinity (*K*_d app_) determinations by only considering protein molecules that are competent for RNP assembly.

Initial binding affinities of crRNA and tracrRNA were characterized by dot-blot as a baseline for comparing inhibitor binding affinities. TracrRNA bound to *Sp*Cas9 with a *K*_d app_ of 10 ± 1 nM, whereas crRNA did not efficiently bind *Sp*Cas9 without tracrRNA present (Supplementary Fig. S1B). In contrast, two separate crRNAs were tested and found to bind with similar affinities to the SpCas9-tracrRNA complex, with *K*_d app_ values of 100 ± 9 nM (crE2) and 114 ± 7 nM (crTR) (Supplementary Fig. S1B). To determine the contribution of base pairing and structural interaction between crRNA and tracrRNA during *Sp*Cas9 RNP assembly, we performed gel-shift assays to measure the binding between tracrRNA and crRNA alone. Quantification of gel shifts revealed very similar binding affinities of 49 ± 5 nM and 41 ± 4 nM for crRNA binding to tracrRNA versus crRNA binding to an *Sp*Cas9-tracrRNA complex, respectively (Supplementary Fig. S2A). While gel-shifts will provide different estimates of binding affinity than equilibrium binding by dot-blots, these results nonetheless suggest that crRNA interaction with the *Sp*Cas9-tracrRNA complex is dominated by base-pairing interactions with the tracrRNA in a dual-guide RNA (dgRNA) system.

After establishing the binding affinity of crRNA and tracrRNA guides during *Sp*Cas9 RNP assembly, we tested the binding affinity of three potential inhibitor modules, Anti1_PAM, Anti1_tracr, and Anti1-guide (Fig. 1C, E). Binding to an *Sp*Cas9-tracrRNA complex by Anti1_PAM exhibited the highest binding affinity, with a *K*_d app_ of 297 ± 21 nM, while Anti1_tracr had an estimated affinity of 980 nM ±189 nM (Fig. 1F). The binding of Anti1_guide to a fully assembled *Sp*Cas9-tracrRNA-crRNA complex was very poor and affinity could not be determined. Binding of anti-tracr modules might be further improved by incorporating other RNA analogs to improve its interaction with Cas9. In contrast, the anti-guide module, which showed little or no binding, might benefit from DNA or DNA analogs to better mimic target DNA. The relatively strong binding of Anti1_PAM is reminiscent of certain natural Acr proteins that interact with the PI domain of Cas9 [29].

**FIG. 2.**
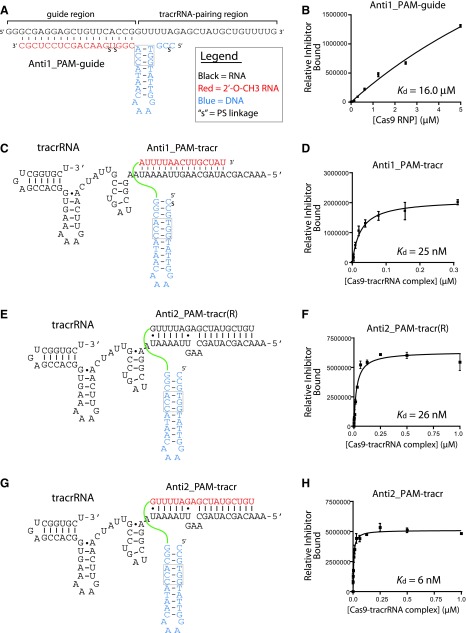
Modular nucleic acid-based inhibitors and their binding to *Sp*Cas9-tracrRNA RNP complex. Sequence, secondary structure, and pairing for each inhibitor design are shown in the *left panels* (**A**, **C**, **E**, and **G**) and the associated binding activity and calculated affinity are shown in the *right panels* (**B**, **D**, **F**, and **H**). Error bars are standard error of the mean (SEM).

Although individual modules did not bind *Sp*Cas9 RNP complexes tightly enough to suggest strong inhibitory potential, we reasoned that improved affinity and specificity might be possible by combining two modules together. We designed an anti-guide fused to an anti-PAM (Anti1_PAM-guide) as a continuous chimeric oligonucleotide (Fig. 2A) to mimic a partial target substrate based on crystal structures of *Sp*Cas9. While binding affinity was better than an anti-guide alone and could be extrapolated to 16.0 ± 4.4 μM (Fig. 2B), it was clear that the anti-guide module might require substantial redesigning and optimization. However, combining the anti-PAM and anti-tracr modules (Anti1_PAM-tracr) by an 18-atom PEG linker resulted in substantially improved binding to an *Sp*Cas9-tracrRNA complex (Fig. 2C, D). The *K*_d app_ for this modular fusion was 25 ± 5 nM, an order of magnitude better than the Anti1_PAM module alone. This result suggested that the proper linking of anti-CRISPR nucleic acid modules could achieve high binding affinity and is likely to improve specificity through multiple points of contact.

The Anti1_tracr module was designed to pair completely to most of the tracrRNA repeat region. An anti-tracr module might achieve better affinity if it mimicked the natural stem structure, which includes a bulge [33]. Thus, we designed a new modular inhibitor that replaced the anti-tracr module with RNA nucleotides to mimic crRNA binding (Fig. 2E). We found that this new design, Anti2_PAM-tracr(R), bound surprisingly well, considering a lack of chemical modifications that usually improve affinity (Fig. 2F). Its *K*_d app_ was 26 ± 3 nM, approximately four times the binding affinity of an average crRNA (Supplementary Figure S1). Indeed, when RNA nucleotides were further replaced with 2′-*O*-methyl in the anti-tracr module (Anti2_PAM-tracr), the binding affinity improved further to 6 ± 1 nM (Fig. 2G, H). Thus, by considering RNP structural features, the binding of candidate inhibitors to *Sp*Cas9-tracrRNA complex can be improved.

**FIG. 3.**
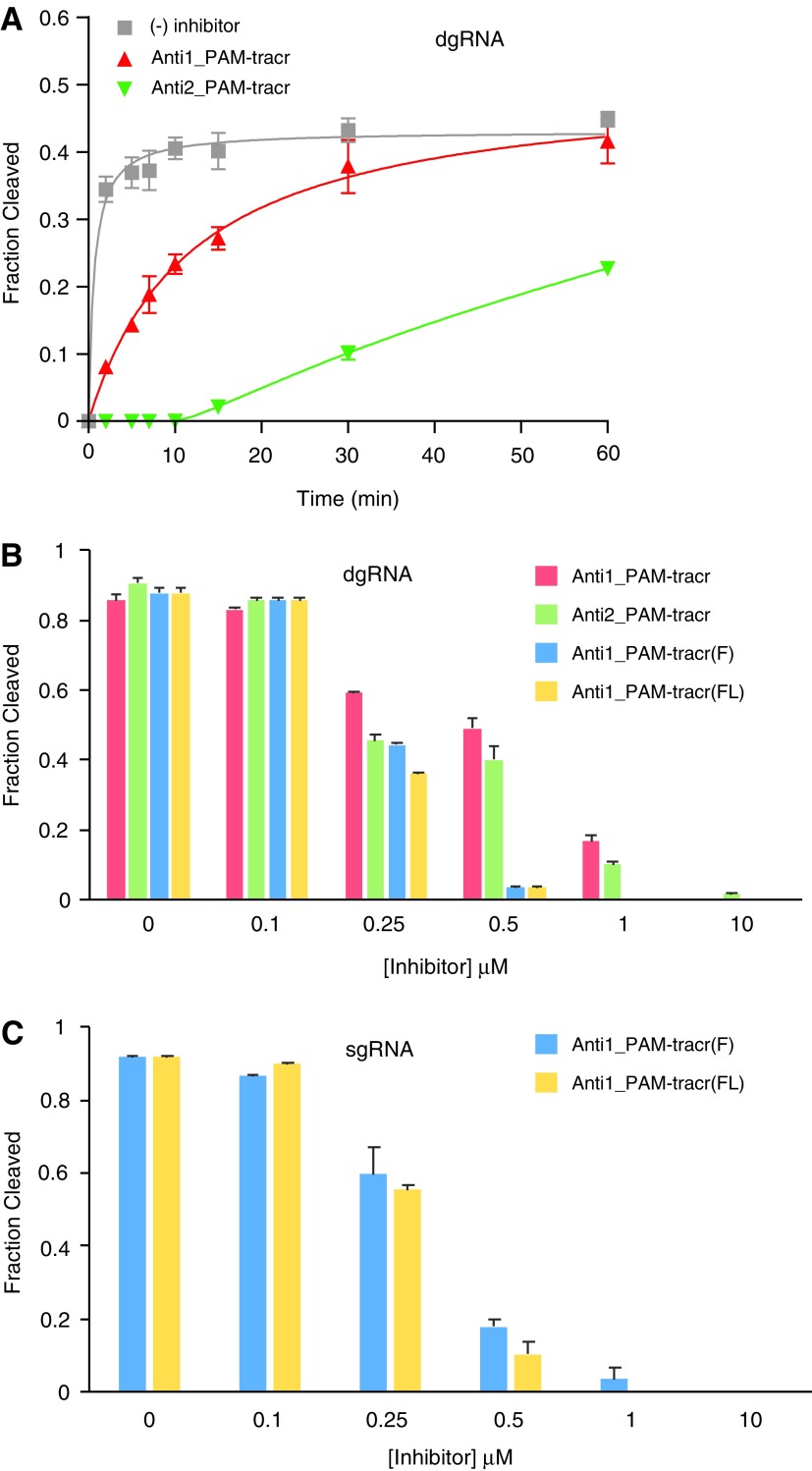
Inhibition of CRISPR-*Sp*Cas9 catalytic activity *in vitro* with nucleic acid-based inhibitors. **(A)** Inhibition of *in vitro Sp*Cas9 cleavage activity over time. Inhibitor and crRNA (crE2) concentrations were set at 1 μM. Error bars are standard error of the mean (SEM). **(B)** Inhibition of 10 min *in vitro* cleavage assay reactions containing 0.25 μM tracrRNA and 0.3 μM crRNA (crEGIP). Error bars are standard error of the mean (SEM). **(C)** Inhibition of the same reaction, but with 0.25 μM sgRNA (sgEGIP). Error bars are standard error of the mean (SEM).

Rational design with improved binding affinities suggested that modular Anti1_PAM-tracr and Anti2_PAM-tracr nucleic acids had the potential to act as anti-CRISPR inhibitors of *Sp*Cas9. To test their effect on enzyme activity, we prepared *in vitro* cleavage reactions. Previous reports had revealed that *in vitro* cleavage reactions proceed very rapidly [35]. To determine the best conditions for evaluating inhibition, we initially tested three conditions (Supplementary Fig. S2B, C). Cas9-tracrRNA complex was assembled first, and then (1) crRNA was incubated to allow full assembly before simultaneously combining with the target DNA and the inhibitor, (2) the crRNA and inhibitor were incubated together with Cas9-tracrRNA to allow direct competition before adding target DNA, or (3) the inhibitor was preincubated to allow stable binding, and then the crRNA and target DNA simultaneously added. In these initial condition tests, two different DNA targets were used, the inhibitor and crRNA were at equimolar final concentrations, and reactions were stopped after 10 min. We found that the last condition, preincubation, slowed the reaction kinetics the most and chose this condition for a time course (Fig. 3A).

The dual RNA-guided enzyme with the particular crRNA (crE2) and linearized EGFP plasmid target used in this reaction are known to only reach ∼50% cleavage *in vitro* [35]. The uninhibited control reaction was 50% completed after 38 ± 11 s. When Anti1_PAM-tracr was present, the reaction slowed substantially, requiring 719 ± 100 s to reach 50% completion. The Anti2_PAM-tracr provided the greatest inhibition under these conditions, with a predicted 50% completion at close to 1 h. These results, combined with binding data, suggest that the crRNA is capable of dynamic interaction with the more stable *Sp*Cas9-tracrRNA complex, which facilitates competitive inhibition. Combining anti-tracr with anti-PAM modules, which creates two distinct binding sites, may be key for achieving strong inhibition and high specificity.

To more rigorously test inhibition, we prepared new *in vitro* cleavage reactions with a PCR-generated target DNA and a new crRNA guide (crEGIP). This reaction typically results in about 90% cleavage activity [36]. In this reaction, tracrRNA was the limiting component at 0.25 μM and crRNA was set at 0.3 μM, creating conditions that replicate a typical *in vitro* cleavage assay [35]. Increasing concentrations of Anti1_PAM-tracr and Anti2_PAM-tracr produced increasing inhibition (Fig. 3B). At 0.25 μM, Anti1_PAM-tracr reduced cleavage to 60%, while Anti2_PAM-tracr reduced cleavage to 45%. Inhibition decreased slightly at 0.5 μM and then substantially by 1 μM. Complete inhibition was observed at 10 μM. At all concentrations tested, Anti2_PAM-tracr appeared to have a slight inhibitory advantage over Anti1_PAM-tracr.

Given the successful inhibition by Anti1_PAM-tracr and Anti2_PAM-tracr, we reasoned that replacing 2′-*O*-methyl modifications in the anti-tracr module with nucleotide analogs capable of increased binding affinity might produce greater inhibition. We synthesized inhibitors with all 2′F-RNA [Anti1_PAM-tracr(F)] or a combination of 2′F-RNA and LNA nucleotides [Anti1_PAM-tracr(FL)] [38–40] (Scheme 1). When titrated into typical *in vitro* cleavage assays, these inhibitors were superior to the 2′-*O*-methyl-containing designs (Fig. 3B). Nearly complete inhibition was observed at 0.5 μM. When an identical experiment was carried out utilizing an sgRNA at 0.25 μM, the Anti1_PAM-tracr(F) and Anti1_PAM-tracr(FL) continued to demonstrate robust inhibition (Fig. 3C). The calculated inhibitory concentration (*IC*_50_) values for Anti1_PAM-tracr(F) and Anti1_PAM-tracr(FL) against a dgRNA were 0.24 and 0.25 μM, respectively, and against an sgRNA were 0.28 and 0.29 μM, respectively (Supplementary Fig. S2D). Thus, with appropriate modification and a modular design strategy, effective nucleic acid-based inhibitors of CRISPR enzymes are possible.

**FIG. 4.**
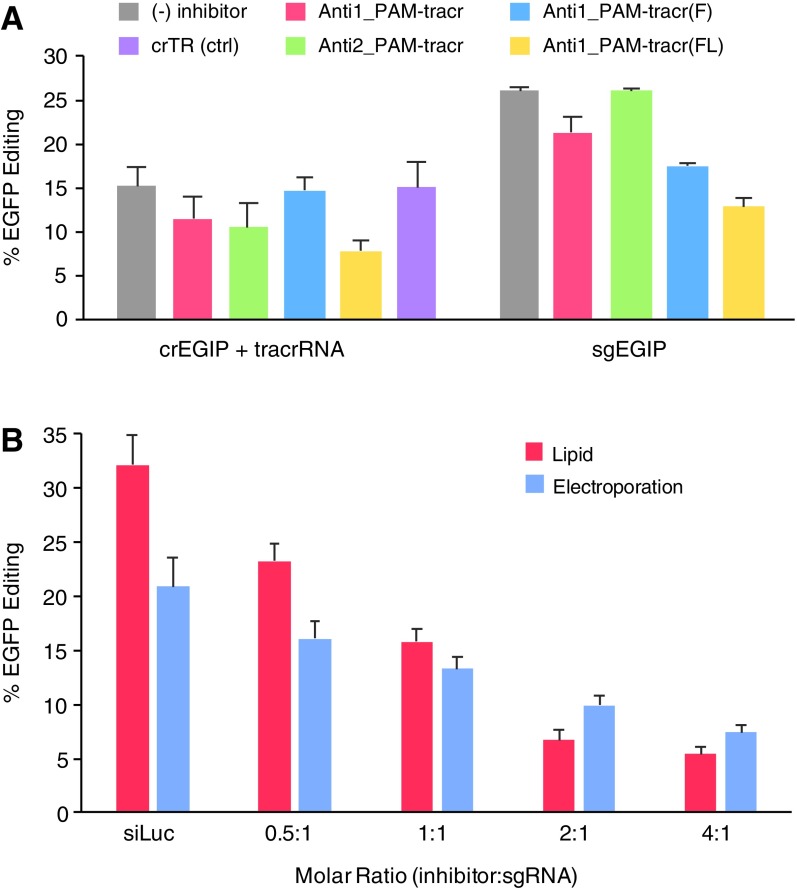
Inhibition of CRISPR-*Sp*Cas9 editing in human cells by nucleic acid-based inhibitors. **(A)** Inhibition of cell-based editing by lipid co-transfection of guide RNAs and inhibitors at a 1:1 molar ratio into HEK 293T cells expressing EGFP and *Sp*Cas9. Editing was measured by flow cytometry. Error bars are standard error of the mean (SEM). **(B)** Dose-dependent inhibition of editing in human cells with sgRNA and inhibitors. sgRNA and inhibitor were lipid co-transfected into HEK 293T cells expressing EGFP and *Sp*Cas9 or sgRNA-Cas9 RNP was co-electroporated into HEK 293T cells expressing EGFP. Error bars are standard error of the mean (SEM). EGFP, enhanced green fluorescent protein.

Application of nucleic acid-based CRISPR-*Sp*Cas9 inhibitors for therapeutics will require efficient inhibition of gene editing inside of human cells. To investigate this possibility, we transfected either dgRNA (tracrRNA with crRNA) or sgRNA into HEK 293T cells stably expressing *Sp*Cas9 and EGFP. The EGFP target sequence was the same as that tested for *in vitro* cleavage with crEGIP [37]. Successful editing should result in loss of EGFP fluorescence. We co-transfected guide RNA with equal amounts of inhibitor and then performed flow cytometry to count EGFP-expressing cells (Fig. 4A). Editing by dgRNA was mildly inhibited by Anti1_PAM-tracr and Anti2_PAM-tracr. In contrast, Anti1_PAM-tracr(F) did not provide substantial inhibition. Anti1_PAM-tracr(FL), however, provided about 50% inhibition. Surprisingly, the sgRNA editing reactions showed improved inhibition by Anti1_PAM-tracr(F) and also 50% inhibition with the Anti1_PAM-tracr(FL) design. The nature of a sgRNA should in principle provide a more difficult target for inhibition due to strong intramolecular base pairing induced by the GNRA tetraloop [2].

Encouraged by these results, we performed transfections testing increasing concentrations of Anti1_PAM-tracr(FL) (Fig. 4B). The observed editing decreased in a dose-dependent manner as the molar ratio of co-transfected inhibitor to sgRNA increased. Because most cell lines do not stably express Cas9, we carried out a similar experiment but co-electroporated the Cas9-sgRNA RNP with Anti1_PAM-tracr(FL) into HEK 293T cells expressing EGFP. Although overall EGFP editing was lower by this method, a similar dose-dependent trend of inhibition was observed. Interestingly, the decrease in editing was not as sharp as in the lipid transfection. Together, these results demonstrate that Anti1_PAM-tracr(FL) effectively inhibits Cas9 in a concentration-dependent manner by multiple methods of delivery in human cells.

## Discussion

Molecules with the potential to inhibit CRISPR activity and be developed into drugs would significantly improve the safety of CRISPR-based therapeutics. In this study, we rationally designed small nucleic acid-based inhibitors, abbreviated as SNuBs, against CRISPR-Cas9 that could inhibit enzyme activity. Our design strategy is based on multiple points of contact, chemical modification, and the mimicry of natural guide RNA or target DNA interactions with CRISPR effector enzymes. We characterized binding modules and found that combining them through linkers improved binding affinity and enzyme inhibition. Having multiple points of interaction should presumably increase the specificity of inhibition as well.

Effective CRISPR SNuBs did not engage the guide sequence, making these inhibitors sequence independent and broadly applicable with respect to targets. Targeting the guide sequence of CRISPR enzymes would necessitate the synthesis and possible optimization of new inhibitors for every target. For some CRISPR enzymes, guide targeting should be avoided. For example, Cas12a (Cpf1) enzymes unleash nonsequence-specific single-stranded DNase activity when their guide is bound to a DNA target or target mimics [41]. Thus, guide-targeted inhibitors could create dangerous off-target cleavage of the genome when single-stranded DNA is accessible, such as during transcription [42], replication [43], and genome repair [44].

Surprisingly, 2′F and 2′F/LNA modified inhibitors also reduced activity of a sgRNA-Cas9 RNP complex *in vitro* and inside cells. The nature of sgRNAs would be expected to preclude access of an anti-tracr module, which would have to displace the 5′ portion of the sgRNA and interrupt intramolecular folding. However, the results presented in this study argue that guide RNA binding by Cas9 is dynamic. Indeed, previous FRET-based measurements identified large Cas9 conformational changes upon sgRNA binding and support the presence of dynamic Cas9-guide RNA interaction [45]. Thus, Cas9 RNP complexes likely possess a greater degree of conformational flexibility and guide RNA exchange potential than previously anticipated, thereby facilitating binding by CRISPR SNuBs.

The modular inhibitors described in this study should be amenable to rational optimization by independently modifying each module, such as length, sequence, and chemistry, and connecting them in a systematic combinatorial manner. Anti-tracr modules should benefit from mimicking the natural RNA structure and incorporating RNA analogs with high binding affinity, such as 2′F-RNA, 2′-methoxyethyl, LNA, or other bicyclo or bridged nucleic acids (BNAs) [39,46]. Anti-PAM modules might benefit from different sequence and structural designs, as well as chemical modifications that mimic DNA and improve nuclease resistance, like arabinonucleic acid (ANA), 2′F-ANA, and alpha-L-LNA [39,46]. Stability against nucleases can be further enhanced by incorporating phosphorothioate linkages in the anti-tracr and anti-PAM modules [47]. Likewise, the chemical nature and length of the linkers that connect modules can also be systematically investigated. Additional modules might even be added to further improve other pharmacologic properties, such as cellular delivery and localization.

The strategy described in this study should conceivably extend to many nucleic acid-guided CRISPR effector enzymes, including Cas9, Cas12a, multicomponent CRISPR enzymes (eg, the Cascade complex), and enzymes from other CRISPR classes and enzyme types [48,49]. Certain designs may also prove effective in inhibiting the DNA binding activity, and therefore function, of catalytically inactive CRISPR effectors, such as “dead” Cas9 [5].

CRISPR SNuBs may present a more immediate path toward development for therapeutic applications than other alternatives. Small molecule inhibitors are still difficult to rationally design and can require substantial time to identify by screening or to optimize with medicinal chemistry [50]. Nucleic acid inhibitors are significantly smaller than known natural anti-CRISPR proteins [27,28] and should benefit from the lessons learned during therapeutic development of antisense oligonucleotides and small interfering RNAs [51], some of which have been recently FDA approved [52,53]. Rational design of SNuBs will also benefit from the growing availability of CRISPR-Cas structural and biochemical data [32], molecular modeling [54,55], and chemical modifications for tuning therapeutic nucleic acid properties [51].

The potential uses for CRISPR SNuBs may extend beyond catalytic mechanisms or therapeutics. Inhibitors might also be useful for attenuating CRISPR inhibition (CRISPRi) or activation (CRISPRa) [5], base editing [56], or other CRISPR-based applications that involve target binding. Natural CRISPR systems in pathogenic bacteria could be specifically disrupted to sensitize them to natural or artificial bacteriophages [57]. They could also provide the basis for emergency control measures for potentially dangerous applications like CRISPR-dependent gene drives [58].

## Supplementary Material

Supplemental data
