# Supplementary material for: Rationally Designed Anti-CRISPR Nucleic Acid Inhibitors of CRISPR-Cas9
Source: Nucleic Acid Ther. 2019 May 30;29(3):136–47. doi: 10.1089/nat.2018.0758 (PMC6555185; doi:10.1089/nat.2018.0758)
Supplement: Supplemental data [file Supp_Data.pdf]

Supplemental Data

Rationally Designed Anti-CRISPR Nucleic Acid Inhibitors of CRISPR-Cas9

Christopher L. Barkau[1], Daniel O'Reilly[2], Kushal J. Rohilla[1], Masad J. Damha[2,*], and Keith T. Gagnon[1,3,*]

[1]Department of Biochemistry & Molecular Biology, School of Medicine, Southern Illinois University
[2]Department of Chemistry, McGill University
[3]Department of Chemistry & Biochemistry, Southern Illinois University
**Corresponding authors:* masad.damha@mcgill.ca, ktgagnon@siu.edu

Table S1: Sequence of RNA and DNA Oligonucleotides

Guide RNAs and Oligonucleotides	Sequence (lowercase = RNA, uppercase = DNA)
crE2	uaccagcaaaacacuccgauguuuuagagcuaugcuguuuug
crTR	gcagagccagccuucuuauuguuuuagagcuaugcuguuuug
crEGIP	gggcgaggagcuguucaccgguuuuagagcuaugcuguuuug
tracrRNA	aaacagcauagcaaguuaaaauaaggcuaguccguuaucaacuugaaaaaguggcaccgagucggugcuu
sgEGIP	gggcgaggagcuguucaccgguuuuagagcuagaaauagcaaguuaaaauaaggcuaguccguuaucaacuugaaaaaguggcaccgagucggugcu
T7 promoter oligo	TAATACGACTCACTATA
T7_tracrRNA_as	AAGCACCGACTCGGTGCCACTTTTTCAAGTTGATAACGGACTAGCCTTATTTTAACTTGCTATGCTGTTTCCCTATAGTGAGTCGTATTA
T7_sgEGIP_as	AAGCACCGACTCGGTGCCACTTTTTCAAGTTTGATAACGGACTAGCCTTATTTTAACTTGCTATTTCTAGCTCTAAAACCGGTGAACAGCTCCTCGCCCTATAGTGAGTCGTATTA
pEGIP_vitro targ_F	ATGCGATGGAGTTTCCCCA
pEGIP_vitro targ_R	CCGCTTTACTTGTACAGCTCG

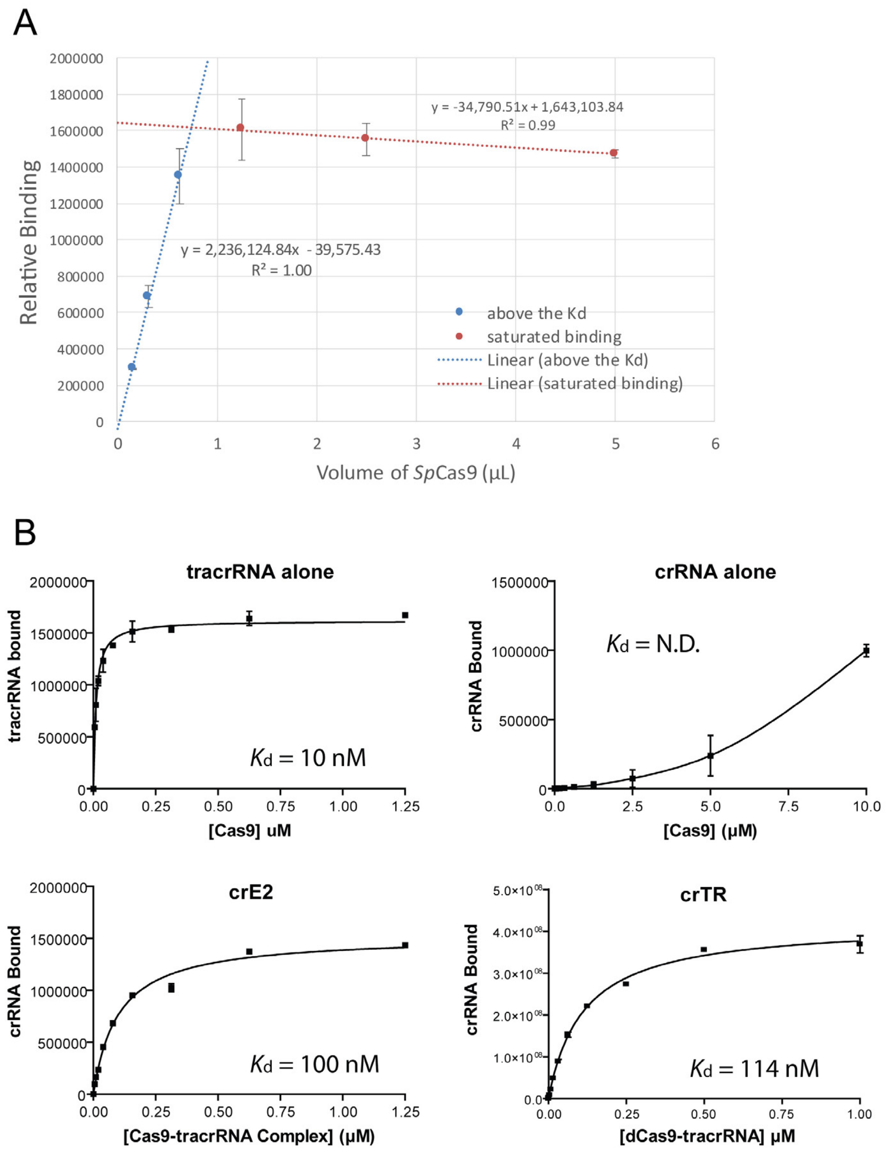

Figure S1. Determining the active concentration of Cas9 and its binding affinity to native dual-RNA guides. (**A**) Cas9 enzyme stocks were titrated with a fixed concentration of tracrRNA-crRNA complex (0.5 µM). Active concentration of Cas9 was determined by solving for x when the equations of the two lines were set equal to each other, as described in Methods. (**B**) Binding of Cas9 to tracrRNA or Cas9-tracrRNA complex to crRNA. Binding affinities were calculated by non-linear regression and fitting to a one-site binding curve. Error bars are standard error of the mean (s.e.m.).

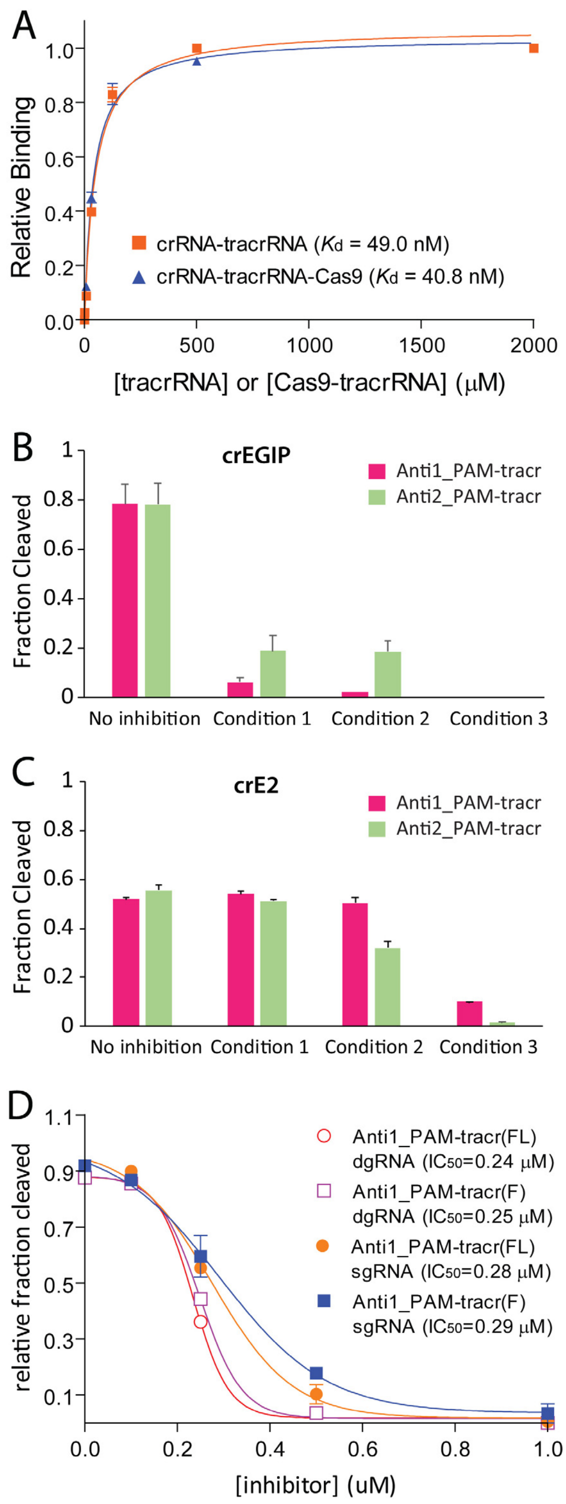

Figure S2. (**A**) Quantification of gel shift analyses comparing crRNA binding and affinity to Cas9-tracrRNA complex or tracrRNA alone. (**B-C**) Testing of different conditions for inhibitor testing in in vitro cleavage assays. Condition 1: Pre-assemble RNP complex, then mix with target and inhibitor, Condition 2: Pre-assemble RNP, incubate with inhibitor, then mix with target, Condition 3: Pre-incubate inhibitor with Cas9-tracrRNA complex, then mix with crRNA and target. (**D**) Quantification of dgRNA and sgRNA Cas9 inhibition. IC_{50} values were calculated by fitting to a variable slope dose-response inhibition curve. All error bars are standard error of the mean (s.e.m.).
